# Estimation of plasma fibrinogen levels based on hemoglobin, base excess and Injury Severity Score upon emergency room admission

**DOI:** 10.1186/cc12816

**Published:** 2013-07-12

**Authors:** Christoph J Schlimp, Wolfgang Voelckel, Kenji Inaba, Marc Maegele, Martin Ponschab, Herbert Schöchl

**Affiliations:** 1 Ludwig Boltzmann Institute for Experimental and Clinical Traumatology, AUVA Research Centre, Donaueschingenstrasse 13, 1200 Vienna, Austria; 2 Department of Anaesthesiology and Intensive Care Medicine, AUVA Trauma Centre, Dr. Franz Rehrl Platz 5, 5020 Salzburg, Austria; 3 Department of Surgery, Los Angeles County and University of Southern California Medical Center, 2051 Marengo Street, Los Angeles, CA 90033, USA; 4 Department of Trauma and Orthopedic Surgery, Institute for Research in Operative Medicine (IFOM), University of Witten/Herdecke, Cologne-Merheim Medical Center (CMMC), Ostmerheimerstrasse 200, 51109 Cologne, Germany; 5 Department of Anaesthesiology and Intensive Care Medicine, AUVA Trauma Centre, Garnisonstrasse 7, 4017 Linz, Austria

## Abstract

**Introduction:**

Fibrinogen plays a key role in hemostasis and is the first coagulation factor to reach critical levels in massively bleeding trauma patients. Consequently, rapid estimation of plasma fibrinogen (FIB) is essential upon emergency room (ER) admission, but is not part of routine coagulation monitoring in many centers. We investigated the predictive ability of the laboratory parameters hemoglobin (Hb) and base excess (BE) upon admission, as well as the Injury Severity Score (ISS), to estimate FIB in major trauma patients.

**Methods:**

In this retrospective study, major trauma patients (ISS ≥16) with documented FIB analysis upon ER admission were eligible for inclusion. FIB was correlated with Hb, BE and ISS, alone and in combination, using regression analysis.

**Results:**

A total of 675 patients were enrolled (median ISS 27). FIB upon admission correlated strongly with Hb, BE and ISS. Multiple regression analysis showed that Hb and BE together predicted FIB (adjusted R^2 ^= 0.46; log_e_(FIB) = 3.567 + 0.223.Hb - 0.007.Hb^2 ^+ 0.044.BE), and predictive strength increased when ISS was included (adjusted R^2 ^= 0.51; log_e_(FIB) = 4.188 + 0.243.Hb - 0.008.Hb^2 ^+ 0.036.BE - 0.031.ISS + 0.0003.ISS^2^). Of all major trauma patients admitted with Hb <12 g/dL, 74% had low (<200 mg/dL) FIB and 54% had critical (<150 mg/dL) FIB. Of patients admitted with Hb <10 g/dL, 89% had low FIB and 73% had critical FIB. These values increased to 93% and 89%, respectively, among patients with an admission Hb <8 g/dL. Sixty-six percent of patients with only a weakly negative BE (<−2 mmol/L) showed low FIB. Of patients with BE <−6 mmol/L upon admission, 81% had low FIB and 63% had critical FIB. The corresponding values for BE <−10 mmol/L were 89% and 78%, respectively.

**Conclusions:**

Upon ER admission, FIB of major trauma patients shows strong correlation with rapidly obtainable, routine laboratory parameters such as Hb and BE. These two parameters might provide an insightful and rapid tool to identify major trauma patients at risk of acquired hypofibrinogenemia. Early calculation of ISS could further increase the ability to predict FIB in these patients. We propose that FIB can be estimated during the initial phase of trauma care based on bedside tests.

## Introduction

Trauma-induced coagulopathy (TIC) has been reported in 25 to 35% of all injured patients upon admission to the emergency room (ER) [1,2]. Established TIC strongly increases the risk of massive transfusion (MT), prolonged length of intensive care unit (ICU) and hospital stay, and mortality [1]. Importantly, coagulation factors do not decrease in a uniform manner in severely bleeding patients. Plasma fibrinogen (FIB) reaches critically low levels earlier than any other coagulation factor [3] and low FIB is almost always the primary coagulation factor deficiency during major bleeding, as shown for example in trauma [4] and postpartum hemorrhage (PPH) [5].

Fibrinogen plays a central role in primary and secondary hemostasis [6]. It has a high affinity for glycoprotein IIb/IIIa receptors which are expressed on activated platelets and is therefore essential for platelet aggregation. Moreover, fibrinogen is a substrate of the coagulation process and precursor of the fibrin network. Low FIB has been associated with increased blood loss and/or transfusion requirements in a number of settings, including cardiac surgery, PPH and trauma [7-11], and has recently been reported to worsen outcomes in trauma patients receiving MT [12]. Thus, early replacement of fibrinogen is beneficial in trauma patients experiencing severe bleeding [13-18], and the recent European trauma guidelines recommend a FIB threshold concentration for bleeding patients of 150 to 200 mg/dL as a trigger for replacement therapy [19].

In the AUVA Trauma Centre of Salzburg, rapid point-of-care (POC) estimation of fibrin polymerization (that is via the FIBTEM assay on thromboelastometry (ROTEM)) has been established to diagnose fibrinogen deficiencies and to guide fibrinogen substitution in major trauma patients [10,14,15,20-22]. POC blood gas analyses and full blood cell counts are routinely performed in severe trauma patients upon admission to the ER. Furthermore, standard laboratory tests including prothrombin time (PT), activated partial thromboplastin time (aPTT) and FIB, are performed in the hospital's central laboratory with the highest priority. A major problem with these tests is the long turnaround time: the average time for results to reach the clinician is typically around 45 minutes and longer durations have been published [23,24]. Furthermore, PT and aPTT were principally designed for monitoring warfarin and heparin. For these reasons, coagulation management in our institution is based mainly on the results of POC tests, particularly ROTEM analysis (thrombelastography (TEG) could be a viable alternative to ROTEM). However, in many trauma centers, neither ROTEM nor TEG is routinely used for assessing trauma patients, and it is also common for FIB to be missing from routine coagulation assessment.

We therefore conducted a retrospective study to investigate the predictive ability of the laboratory parameters hemoglobin (Hb) and base excess (BE) to estimate FIB of major trauma patients upon admission to the ER. To increase the accuracy of these predictions, we included the Injury Severity Score (ISS) in the analyses.

## Material and methods

The ethics committee of the federal state of Salzburg (Ehtikkommission für das Bundesland Salzburg) approved (Protocol: 415-EP/73/197-2013) this retrospective analysis of data from all major trauma patients (ISS ≥16) admitted to the ER of the AUVA Trauma Centre, Salzburg, Austria, between January 2005 and December 2012 and waived the need for patients' informed consent.

The laboratory parameters of interest in this study, Hb (g/dL), BE (mmol/L) and FIB (mg/dL, Clauss method) were documented with the time of admission in the electronic patient database of the hospital or the patient charts. We considered the main parameters for quick estimation of FIB in this study to be Hb and BE. However, for the addition of a measure of injury severity, we also aimed to include ISS.

ISS, hospital mortality, sex and age were documented in the electronic ICU documentation (ICdoc) or, in cases where the patient was not admitted to the ICU, in the electronic patient database of the hospital. ISS was calculated according to the final diagnosis of all detected patient injuries at the end of stay.

### Statistical analysis

Continuous study variables (FIB, Hb, BE, ISS and age) were analyzed for normal distribution by the Kolmogorov-Smirnov test. To detect differences between groups, either the Student's *t *test or the Mann-Whitney *U *test was performed, depending on the underlying distribution. Categorical variables were analyzed with Fisher's exact test. Overall group differences were compared by the Kruskal-Wallis or the chi-square test. A Spearman correlation analysis was performed to correlate Hb, BE and ISS with FIB. Unless otherwise stated, data are presented as median (interquartile range (IQR)) for continuous variables, and as number (percentages) for categorical variables. Overall, a *P *value <0.05 was considered significant for all statistical tests. Standard statistical calculations were performed using GraphPad Prism 5 (GraphPad Software, La Jolla, CA, USA).

A multivariate analysis was carried out to examine the ability of the parameters Hb, BE and ISS to predict FIB. Linear regression was used to examine the relationship between all three parameters and FIB. Due to the slightly positively skewed distribution and issues of homoscedasticity, the regression analyses were performed with FIB on the log scale. Initially, the effect of each predictor variable upon FIB was examined separately, and the nature of the relationship between each predictor and the outcome was examined. If the relationship was found to be nonlinear, then squared terms for each predictor were introduced. Subsequently, the effect of each of the predictor variables was evaluated in combination using predetermined combinations of variables. The predictive ability of the models was assessed by the R^2 ^and adjusted R^2 ^statistics. Multivariate statistical calculations were performed using the statistical software package Stata 12.1 (StatCorp LP, College Station, TX, USA).

## Results

A total of 680 major trauma patients with documented FIB analysis immediately after admission were identified. Of these, 675 were eligible for inclusion (three patients were excluded due to participation in another study, and a further two patients were excluded for nontrauma-related admission). ISS and Hb values were available for all 675 patients, BE for 576 patients only.

Median ISS was 27 (20 to 38 IQR), median age was 45 (27 to 59 IQR) and 537 (79.6%) patients were male. Overall in-hospital mortality of the study cohort was 15.7% (*n *= 106). Patient characteristics were stratified according to ISS (Table 1).

**Table 1:** Patient demographics stratified according to Injury Severity Score.

	ISS 16-24	ISS 25-34	ISS 35-49	ISS 50-75	*P *value for all 4 group differences
**Patients n (% of total)**	242 (35.9%)	249 (36.9%)	97 (14.4%)	87 (12.9%)	N/A
**Age (range)**	42.5 (26-56)	47 (29.5-60)ns	45 (24-59)ns	42 (25-59)ns	nsKruskal- Wallis
**Male n (% of ISS group)**	200 (82.6%)	195 (78.3%)ns	76 (78.4%)ns	66 (75.9%)ns	nsChi-square
**Mortality n (% of ISS group)**	5 (2.1%)	32 (12.9%)***	27 (27.8%)**	42 (48.3%)**	***Chi-square
**FIB mg/dL (IQR)**	235 (182.5-286.3)	191.0 (143-240)***	134 (94.5-185.5)***	114 (69-156)*	***Kruskal- Wallis
**Hb g/dL (IQR)**	13.0 (11.7-14.2)	12.3 (10.6-13.7)***	10.9 (8.4-13.0)***	9.6 (7.0-11.6)**	***Kruskal- Wallis
**BE mmol/L (IQR)**	−2.6 (−4.5 to −1.4)	−3.7 (−6.2 to −2.1)***	−5.7 (−8.5 to −2.9)***	−7.1 (−10.9 to −4.6)**	***Kruskal- Wallis

ns, not significant, *P <0.05, **P <0.01, ***P <0.001, significance values for comparison with previous group. BE, base excess; FIB, plasma fibrinogen; Hb, hemoglobin; IQR, interquartile range; ISS, Injury Severity Score.

### Correlation and multiple regression analysis

**Figure 1:**
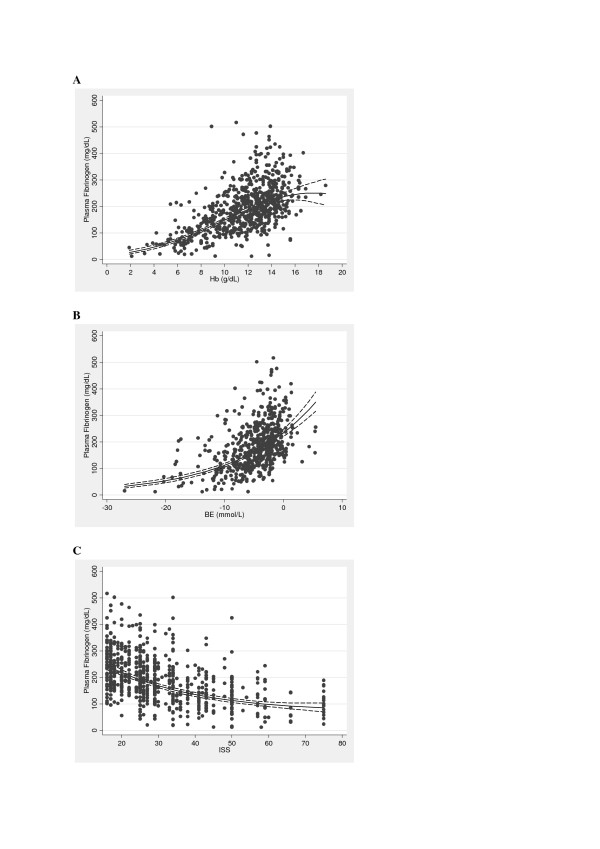
**Correlation between FIB and Hb, BE or ISS**. Plasma fibrinogen concentration (FIB, mg/dL) versus **(A) **hemoglobin (Hb, g/dL), **(B) **base excess (BE, mmol/L) and **(C) **Injury Severity Score (ISS). The fitted regression line (solid) is shown along with a corresponding 95% confidence interval (dotted lines).

When examined individually, all three variables (Hb, BE and ISS) were strongly associated with FIB (Figure 1A to 1C). We then examined the separate effect of each of the three predictor variables upon FIB. The results of these analyses are summarized in Table 2. The strongest predictor was Hb (R^2 ^= 0.40), followed by BE (R^2 ^= 0.29); these parameters explained the largest proportion of the variation in fibrinogen values. The relationship between FIB and the predictor variables Hb and ISS were found to be nonlinear.

**Table 2:** Effect of hemoglobin, base excess and Injury Severity Score upon plasma fibrinogen.

Variable	Term	Ratio (95% CI)	*P *value	R^2^	Adj. R^2^
Hb*	Linear term	4.96 (3.47, 7.11)	<0.001	0.40	0.40
	Squared term	0.80 (0.73, 0.86)			
BE*	Linear term	1.44 (1.38, 1.51)	<0.001	0.29	0.29
ISS**	Linear term	0.70 (0.62, 0.79)	<0.001	0.26	0.25
	Squared term	1.02 (1.00, 1.03)			

*Ratios reported for a 5-unit increase in predictor variable; **ratios reported for a 10-unit increase in predictor variable. Adj., adjusted; BE, base excess; Hb, hemoglobin; ISS, Injury Severity Score.

Subsequently, various combinations of variables were analyzed together in a series of multiple regression analyses. The results of these analyses are summarized in Table 3. Even after adjusting for the effects of the other variables each of the three variables remained statistically significant, suggesting that all three are independent predictors of FIB.

**Table 3:** Multiple regression models showing the combined effect of hemoglobin, base excess and Injury Severity Score upon plasma fibrinogen.

Model	Variable	Term	Ratio (95% CI)	*P *value	R^2^	Adj. R^2^
1	Hb*	Linear term	3.21 (2.20, 4.68)	<0.001	0.47	0.46
		Squared term	0.84 (0.78, 0.92)	<0.001		
	BE*	Linear term	1.25 (1.19, 1.30)			
2	BE*	Linear term	1.33 (1.26, 1.39)	<0.001	0.38	0.38
	ISS**	Linear term	0.71 (0.63, 0.80)	<0.001		
		Squared term	1.03 (1.01, 1.04)			
3	Hb*	Linear term	4.49 (3.19, 6.34)	<0.001	0.47	0.47
		Squared term	0.79 (0.73, 0.86)			
	ISS**	Linear term	0.75 (0.68, 0.83)	<0.001		
		Squared term	1.02 (1.01, 1.04)			
4	Hb*	Linear term	3.37 (2.34, 4.87)	<0.001	0.51	0.51
		Squared term	0.83 (0.76, 0.90)			
	BE*	Linear term	1.20 (1.14, 1.25)	<0.001		
	ISS**	Linear term	0.73 (0.66, 0.82)	<0.001		
		Squared term	1.03 (1.02, 1.04)			

*Ratios reported for a 5-unit increase in predictor variable; **ratios reported for a 10-unit increase in predictor variable. Adj., adjusted; BE, base excess; CI, confidence interval; Hb, hemoglobin; ISS, Injury Severity Score.

By analyzing different combinations of the three variables, the following models for the prediction of FIB were generated:

Model 1 (Hb, BE) log_e_(FIB) = 3.567 + 0.223.Hb - 0.007.Hb^2 ^+ 0.044.BE

Model 2 (BE, ISS) log_e_(FIB) = 6.129 + 0.056.BE - 0.034.ISS + 0.0003.ISS^2^

Model 3 (Hb, ISS) log_e_(FIB) = 3.609 + 0.301.Hb - 0.009.Hb^2 ^- 0.029.ISS + 0.0002.ISS^2^

Model 4 (Hb, BE, ISS) log_e_(FIB) = 4.188 + 0.243.Hb - 0.008.Hb^2 ^+ 0.036.BE - 0.031.ISS + 0.0003.ISS^2^

Model 1, including the two main variables to quickly calculate FIB, provides an adjusted R^2 ^value of 0.46, suggesting that 46% of the variation in fibrinogen values can be explained by the combination of Hb and BE (Table 3). The model containing all three variables (Model 4) provides the highest predictive ability. The adjusted R^2 ^value from this model was 0.51, suggesting that 51% of the variation in fibrinogen values can be explained by the combination of three predictor variables.

### Clinical applicability

Overall, 74% of major trauma patients admitted with Hb <12 g/dL had low (<200 mg/dL) FIB, and critical (<150 mg/dL) FIB was observed in 54%. Of patients admitted with Hb <10 g/dL, 89% had low FIB and 73% had critical FIB. These values increased to 93% and 89%, respectively, among patients with an admission Hb <8 g/dL. Sixty-six percent of patients with only a weakly negative BE (<−2 mmol/L) showed low FIB. Of patients with BE <-6 mmol/L upon admission, 81% had low FIB and 63% had critical FIB. Changing the criterion to an admission BE <-10 mmol/L produced a low FIB rate of 89% and a critical FIB rate of 78%. Considering the ISS, 56% of major trauma patients with a score ≥16 showed low FIB. The corresponding percentages among patients with ISS ≥25, ≥35 and ≥50 were 68%, 88% and 93%, respectively. Critically low FIB was observed in 67% of patients with ISS ≥35 and in 74% of those with ISS ≥50.

Based on results from the above evaluation, we designed charts to rapidly, easily and accurately identify those patients at risk of low (<200 mg/dL) and critical (<150 mg/dL) FIB levels.

For each of the three variables patients were arranged into the following categories:

Hb (g/dL) <8.0; 8.0 to 9.9; 10.0 to 11.9; ≥12.0

BE (mmol/L) <−10; −10.0 to −6.1; −6.0 to −2.1; ≥−2.0

ISS 16 to 24; 25 to 34; 35 to 49; 50 to 75

**Figure 2:**
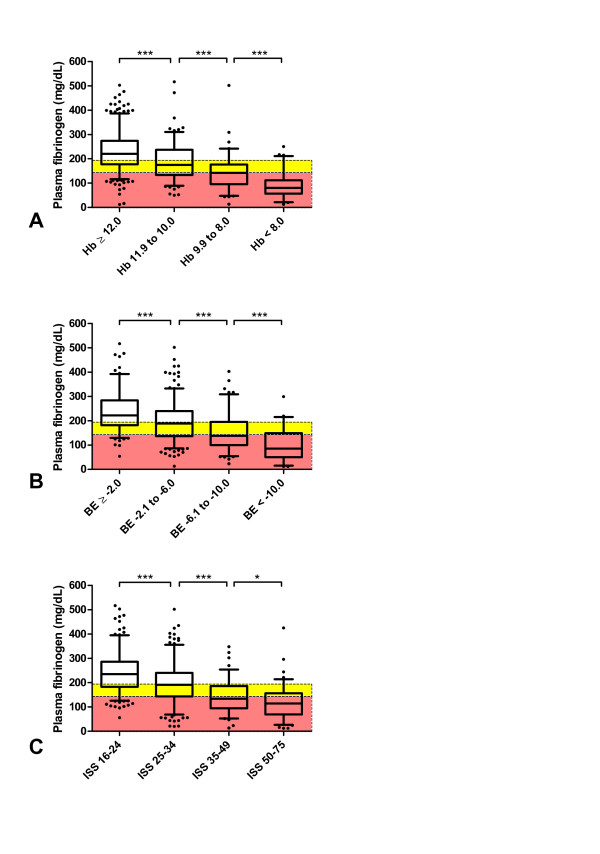
**FIB stratified according to different categories of Hb, BE and ISS**. Plasma fibrinogen concentration (FIB, mg/dL) stratified according to **(A) **hemoglobin (Hb, g/dL), **(B) **base excess (BE, mmol/L) and **(C) **Injury Severity Score (ISS).***P <0.001. Boxes indicate the range between the 25^th ^and 75^th ^percentile, whiskers represent the range between the 5^th ^and 95^th ^percentile, horizontal lines represents the median, and black circles represent outliers. Yellow areas represents FIB values between 199 and 150 mg/dL, red areas indicates FIB values below 150 mg/dL.

FIB was found to significantly change according to each category (Figure 2A to 2C).

**Figure 3:**
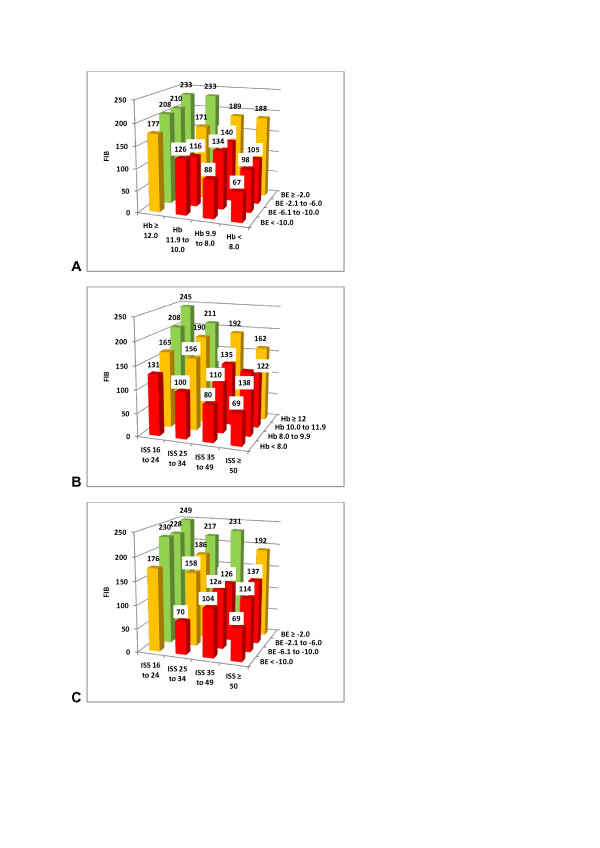
**Combined admission variables resulting in a median plasma fibrinogen level (FIB, mg/dL)**. **(A) **Hemoglobin (Hb, g/dL) and base excess (BE, mmol/L), **(B) **Injury Severity Score (ISS) and Hb, and **(C) **ISS and BE. Green columns indicate adequate FIB (≥200 mg/dL), yellow columns indicate low FIB (<200 mg/dL), and red columns indicate critical FIB (<150 mg/dL).

**Figure 4:**
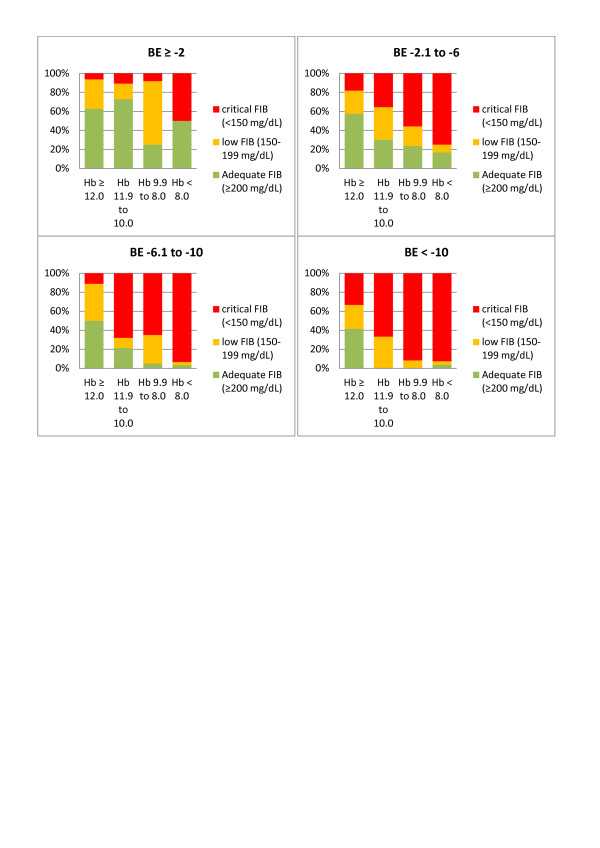
**Percentage of patients with adequate, low and critical FIB stratified according to Hb and BE**. Observed percentage of patients with adequate plasma fibrinogen concentration (FIB) (≥200 mg/dL, green), low FIB (199 to 150 mg/dL, yellow) and critical FIB (<150 mg/dL, red) when combining the admission variables hemoglobin (Hb, g/dL) and base excess (BE, mmol/L).

**Figure 5:**
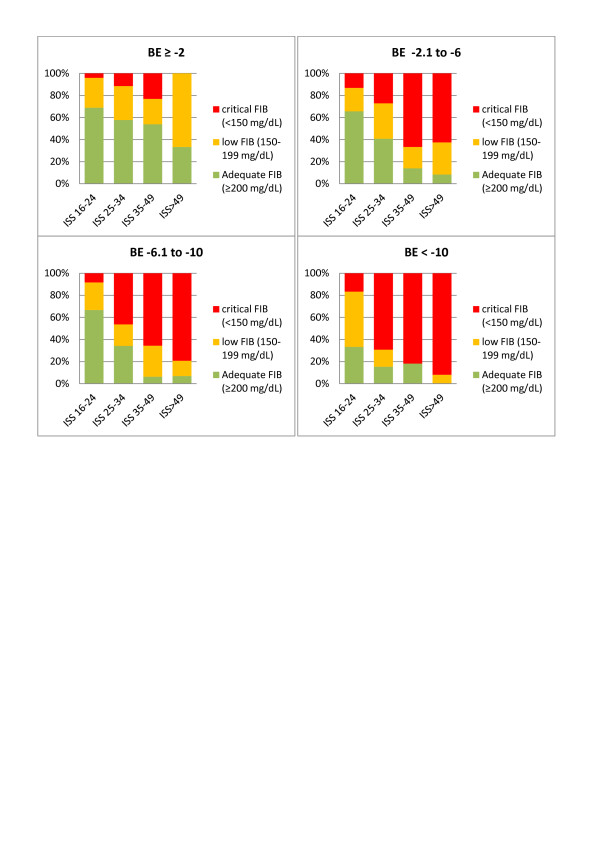
**Percentage of patients with adequate, low and critical FIB stratified according to ISS and BE**. Observed percentage of patients with adequate plasma fibrinogen concentration (FIB) (≥200 mg/dL, green), low FIB (199 to 150 mg/dL, yellow) and critical FIB (<150 mg/dL, red) when combining the admission variables Injury Severity Score (ISS) and base excess (BE, mmol/L).

**Figure 6:**
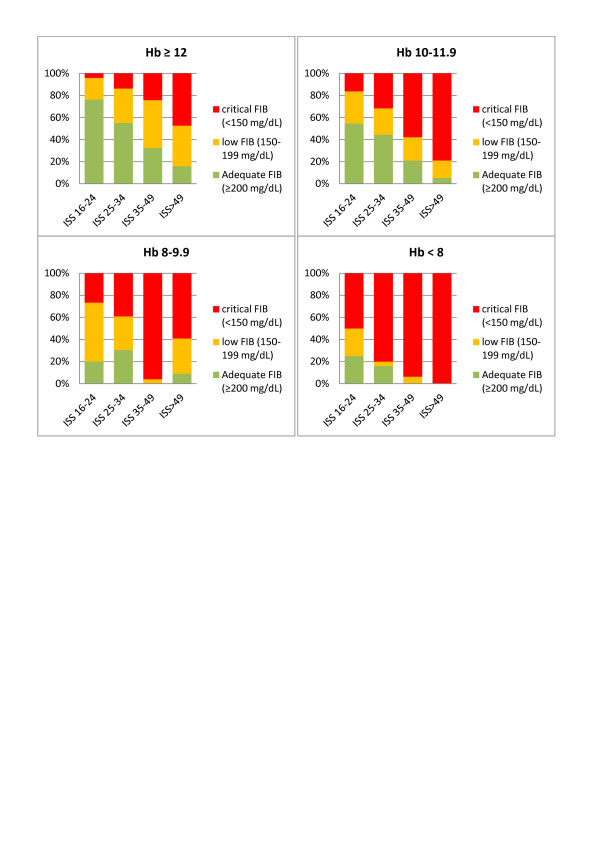
**Percentage of patients with adequate, low and critical FIB stratified according to ISS and Hb**. Observed percentage of patients with adequate plasma fibrinogen concentration (FIB) (≥200 mg/dL, green), low FIB (199 to 150 mg/dL, yellow) and critical FIB (<150 mg/dL, red) when combining the admission variables Injury Severity Score (ISS) and hemoglobin (Hb, g/dL).

We then combined two variables together at a time and calculated the resulting median FIB level for each new group (Figure 3). In this way, we were able to easily identify patients at risk of low or critical FIB, when combining the rapidly obtainable, routine laboratory parameters Hb and BE upon admission (Figure 3A). Combining ISS with either Hb or BE can be used in the same way (Figures 3B and 3C). In each category, the observed percentage of patients with adequate FIB (≥200 mg/dL, green), low FIB (199 to 150 mg/dL, orange) and critical FIB (<150 mg/dL, red) was identified and is shown in Figures 4 to 6.

## Discussion

In the current study, we found that FIB concentration upon admission to the ER is strongly correlated with the severity of injury, blood loss, dilution and shock. The statistical analysis suggests that Hb, BE and ISS, either alone or in combination, allow estimation of FIB in major trauma patients upon admission. Together, these three parameters account for approximately 51% of the variability in the observed fibrinogen concentration values. Furthermore, Hb and BE in combination (and alternatively/additionally ISS) could be used to rapidly identify major trauma patients at risk of low (<200 mg/dL) and critical (<150 mg/dL) FIB concentration.

The most striking finding of the current study is that 51% of the variability in FIB can be explained by Hb, BE and ISS. Shock and substantial tissue injury have been identified as important drivers of TIC, resulting in a profibrinolytic state via the activation of the protein C pathway [1,19,25,26]. Our findings are a proof of the concept that TIC (considered to be a distinct, multifactorial, primary disorder, secondarily amplified by consumption, loss and dilution) requires two conditions: hemodynamic instability (that is shock) and sufficiently massive tissue damage. Therefore, besides blood loss and dilution, both tissue trauma and hypoperfusion independently decrease FIB concentration.

Fibrinogen is an important coagulation factor, acting not only as the precursor of fibrin, the end product of the coagulation process [6,27], but also as a key ligand between activated platelets [28]. In several clinical studies in cardiac surgery and PPH, fibrinogen deficiency was associated with increased blood loss and transfusion requirements [7-9]. For trauma patients, low fibrinogen on admission to the ER was associated with increased transfusion requirements and mortality [10,11]. These data have been confirmed recently by further analyses of the PROMMTT study [29].

There are many reasons for decreased FIB concentration, including easily detectable variables such as blood loss, dilution, hypothermia and acidosis, and harder to detect variables such as consumption, hyperfibrinolysis and measurement-specific variability. In severe bleeding during elective surgery, FIB reaches critically low levels earlier than any other coagulation protein [3]. In light of the threshold levels used by Hiippala *et al*. (100 mg/dL), it may be speculated that current recommended thresholds levels (150 to 200 mg/dL) could be reached with a smaller volume of blood loss.

Rourke *et al*. showed that increasing shock severity was associated with a drop in FIB (*n *= 517) [16]. The same group recently found in a study of 300 patients that TIC, as defined by a calculated prothrombin time ratio >1.2, is associated with a low mean FIB of 96 mg/dL, as compared to 220 mg/dL in noncoagulopathic patients [30]. This suggests that PT is a predictor of coagulopathy caused by low fibrinogen. Floccard *et al*. also reported that in blood samples drawn immediately upon admission from trauma patients with ISS >40, median FIB concentration was 120 mg/dL [31]. Dilutional coagulopathy was unlikely, as patients did not receive significant amounts of fluid prior to blood collection; therefore, low observed FIB levels could be due to profibrinolytic breakdown and/or consumption. These results are in agreement with those from a study by Tauber *et al*. of coagulation factor levels in 334 trauma patients on admission to the ER, in which FIB concentration was significantly reduced (median 160 mg/dL) in patients with ISS >50 [11]. This is similar to our results, where we observed a median FIB of 114 mg/dL for all patients with ISS >50, and higher median FIB concentrations in patient groups with ISS 35 to 49 (median 134 mg/dL), ISS 25 to 34 (median 191 mg/dL) and ISS 16 to 24 (median 235 mg/dL) (Table 1).

Fluid therapy plays an essential role in restoring intravascular volume in massive hemorrhage. The main side effect of volume resuscitation using crystalloids is dilutional coagulopathy. Additionally, artificial colloids, such as starches and gelatins, impair fibrin polymerization [32]. Data from 8,724 patients from the German trauma registry revealed that early traumatic coagulopathy was associated with the amount of intravenous fluids administered preclinically [2]. Floccard *et al*., who studied FIB levels at the trauma scene and in the ER, observed a decrease in median FIB from 260 to 210 mg/dL in patients receiving median 500 mL of prehospital fluid [31]. In more severely injured patients (ISS >40) receiving 1,250 mL of prehospital fluid, a FIB of 120 mg/dL was measured. The fall in FIB levels observed between the trauma scene and admission to the ER may be explained in part by the administration of intravenous fluids. The impact of blood loss and subsequent dilution due to prehospital fluid administration can be detected in our study, as Hb is directly proportional to FIB (Table 2; Figure 1A); this may explain up to 40% of the variation in FIB observed in the studied patients.

In the current study, we have shown that more negative BE values are associated with lower FIB levels. Although current experimental models of acidosis are insufficient to explain trauma and shock-induced acidosis, Martini *et al*. showed that, in a pig model, HCl-induced acidosis resulted in premature degradation of fibrinogen [33]. Furthermore, White *et al*. observed reduced fibrinogen with increasing severity of shock [34]. This suggests a possible cause for the low FIB values in patients with more negative BE values in this study.

Increased consumption of coagulation factors at the site of injury may contribute to lower FIB levels in trauma patients. In patients with severe traumatic brain injury there is an increased risk of disseminated intravascular coagulation (DIC) [21], which would further increase the consumption of coagulation factors. Furthermore, hyperfibrinolysis (HF) is common in trauma and this can lead to very low FIB values. Schöchl *et al*. observed FIB levels of 80 ± 40 mg/dL in 33 trauma patients with established HF diagnosed with ROTEM [20]. Cotton *et al*. reported a median FIB value of 55 (52 to 219 IQR) mg/dL in 41 patients with HF, as diagnosed by TEG [35]. Despite resulting in low FIB values, HF cannot be detected directly by standard laboratory parameters alone [36]. In our current study, HF could have led to some extreme outliers with very low FIB values, even in patient groups not usually at risk of critical FIB levels (Figure 2).

When comparing FIB across different studies, it has to be taken into account that there are more than 60 different ways of measuring fibrinogen concentration, including numerous variations of the von Clauss method [37]. This introduces many factors that may affect the measured FIB, including the type of device, software, readout method, activators or calibration. Furthermore, it has been observed that the presence of artificial colloids (for example dextran or hydroxyethyl starch (HES)) significantly raises FIB measured using the PT-derived and von Clauss methods to levels above those predicted by the dilutional effect [38]. Thus, when high volumes of synthetic colloids are used during MT, hypofibrinogenemia may potentially be overlooked. This possibility has been confirmed by Adam *et al*., who reported that photo-optical methods significantly overestimate FIB in blood diluted with HES [39]. These studies showed that FIB was overestimated by >50% and >100% with 30% and 50% dilution, respectively. Notably, in this context the concentration of HES appears to be more important than molecular size [39,40].

Data showing that fibrinogen supplementation improves survival in trauma patients are limited. However, experimental and initial clinical studies have reported promising outcomes following replacement of fibrinogen as the initial step in managing TIC [13,15,41,42]. Stinger *et al*. were the first to show that a high ratio of red blood cells (RBCs) and fibrinogen resulted in improved outcomes [13]. Currently there are three potential sources of fibrinogen replacement therapy: therapeutic plasma (such as fresh frozen plasma (FFP)), cryoprecipitate and fibrinogen concentrate (FC). When using FFP, high-volume transfusion is necessary to provide sufficient increase in FIB. Larger and more rapid increases can be achieved using either cryoprecipitate or FC. However, cryoprecipitate contains variable quantities of fibrinogen, it must be thawed before use, and there have been significant safety concerns regarding the use of this product, mainly relating to a lack of viral inactivation. In contrast, FC is rapidly available, contains a high and standardized amount of fibrinogen, and is virally inactivated. In most European countries, cryoprecipitate is no longer used, and instead FC is available, delivering consistent amounts of fibrinogen [43]. Currently, a randomized pilot study in trauma patients is exploring the use of FC in the prehospital area [44].

The main limitation of the present study is its retrospective, uncontrolled nature. For example, we were not able to identify co-medications or comorbidities that could have influenced test results. Furthermore, we did not document hypothermia, which has been shown to influence fibrinogen synthesis in the liver [33]. However, this seems to be of greater importance for a longer observation period rather than upon ER admission. We also did not document preclinical fluid therapy, which might significantly influence TIC [2] and therefore FIB upon admission. However, we speculate that the admission parameter Hb indirectly includes the influence of preclinical fluid therapy on FIB levels. The multiple regression model of Hb, BE and ISS explains around 51% of the variation observed in admission FIB. While the remaining variation is still unexplained by these variables, estimation of FIB through Hb, BE and ISS seems acceptable and inclusion of further variables is unlikely to substantially improve prediction. A high percentage of variation may arise from the normal physiological variation seen in human FIB values (200 to 450 mg/dL) [10]. Patients with high normal baseline FIB values will tolerate much higher blood loss and dilution before FIB falls to critical levels, as compared to patients in the lower range of normal baseline values, in whom even a little hemodilution may induce dilutional coagulopathy [45]. Another consideration is that ISS is normally calculated at the end of stay, limiting the possibility of using this parameter for an early estimate of FIB. However, the ISS was developed in 1974 when trauma scans were not universally available [46]. Nowadays, practically all trauma centers in industrialized countries are equipped with computed tomography (CT) scanners, enabling important injuries to be diagnosed within a couple of minutes. Thus, an early estimation of ISS could conceivably be used for estimating FIB. Finally, we only included patients with blunt trauma. Therefore, the results of the current study provide FIB estimation for blunt injury only and will need to be confirmed in other trauma settings.

## Conclusions

Upon ER admission, FIB of major trauma patients shows strong correlations with rapidly obtainable, routine laboratory parameters such as Hb and BE. These two parameters might provide an insightful and rapidly available tool to identify major trauma patients at risk of acquired hypofibrinogenemia. Early calculation of ISS can further increase the ability to predict FIB in these patients. We propose that FIB can be estimated during the initial phase of trauma care based on bedside tests.

## Key messages

• Plasma fibrinogen of major trauma patients shows strong correlation with rapidly obtainable, routine laboratory parameters such as hemoglobin and base excess, upon admission to the emergency room.

• Based on clinical data, these two parameters might provide an insightful and rapidly available tool to identify major trauma patients at risk of acquired hypofibrinogenemia.

• Early calculation of Injury Severity Score can further increase the ability to predict plasma fibrinogen in these patients.

## Abbreviations

aPTT: activated partial thromboplastin time; BE: base excess; CT: computed tomography; DIC: disseminated intravascular coagulation; ER: emergency room; FC: fibrinogen concentrate; FFP: fresh frozen plasma; FIB: plasma fibrinogen; Hb: hemoglobin; HF: hyperfibrinolysis; HES: hydroxyethyl starch; ICU: intensive care unit; IQR: interquartile range; ISS: Injury Severity Score; MT: massive transfusion; POC: point-of-care; PPH: postpartum hemorrhage; PT: prothrombin time; RBCs: red blood cells; ROTEM: thromboelastometry; TEG: thrombelastography; TIC: trauma-induced coagulopathy.

## Competing interests

Christoph Schlimp has received research support from CSL Behring. Wolfgang Voelckel declares no conflicts of interest. Kenji Inaba declares no conflicts of interest. Marc Maegele declares no conflicts of interest. Martin Ponschab declares no conflicts of interest. Herbert Schöchl has received speaker honoraria and research support from CSL Behring.

## Authors' contributions

CJS and HS conceived the study design, collected, analyzed and interpreted the data, performed the statistical analysis and wrote the manuscript. MP contributed substantially to collecting and interpreting the data and critically revised the manuscript for important intellectual content. WV, KI and MM contributed substantially to interpreting the data and critically revised the manuscript for important intellectual content. All authors read and approved the final manuscript.
